# Complete Genome Sequence of *Potato Virus T* from Bolivia, Obtained from a 33-Year-Old Sample

**DOI:** 10.1128/MRA.01066-18

**Published:** 2018-11-08

**Authors:** Ian P. Adams, Jorge Abad, Cesar E. Fribourg, Neil Boonham, Roger A. C. Jones

**Affiliations:** a Fera Science Ltd, Sand Hutton, York, United Kingdom; b Departamento de Fitopatologia, Universidad Nacional Agraria, La Molina, Lima, Peru; c School of Natural and Environmental Sciences, Newcastle University, Newcastle upon Tyne, United Kingdom; d Institute of Agriculture, Faculty of Science, University of Western Australia, Crawley, Western Australia, Australia; e Department of Primary Industries and Regional Development, South Perth, Western Australia, Australia; University of Delaware

## Abstract

We present the complete genomic sequence of a *Potato virus T* (PVT) isolate originally obtained from a Bolivian potato sample collected in 1976, and we compare it with the genome of the PVT type isolate from Peru. There is an 81% nucleotide identity between the two genomes of this Andean potato virus.

## ANNOUNCEMENT

In 1976, tuber sample OCH-10488 was obtained from subsistence potato plantings (Solanum tuberosum subsp. andigena) in the Bolivian Andes and sent to Lima, Peru. *Potato virus T* (PVT; proposed genus Tepovirus, family *Betaflexiviridae*) was detected in plants grown from it using serological tests and inoculation to indicator hosts. The isolate obtained (PVT-Bol) infected 25 indicator hosts belonging to 8 families and was transmitted through seed to seedlings of Chenopodium quinoa and Nicotiana debneyi (1). PVT was first isolated in Peru in 1972 and subsequently described as a new Andean potato virus (2–4). In 1979, infected leaves were dried over silica gel, sealed in glass vials, and sent to England, where PVT-Bol was transmitted to potato seedlings via seed from both infected plants and healthy plants pollinated with infected plant pollen (5). The transmission of PVT through potato botanical seed constitutes a pathway for its inadvertent introduction to other continents (5, 6). In 1985, leaves infected with PVT-Bol were freeze-dried in glass vials and kept in the plant virus collection at Fera Science Ltd (York, England). More recently, a complete genome of the PVT type isolate from Peru (PVT-Type, GenBank accession number NC_011062) was described (2–4, 7).

In 2017, total RNA was extracted from freeze-dried leaf material infected with PVT-Bol using an RNAeasy kit (Qiagen, UK) including optional DNase treatment. An indexed, plant ribosome-subtracted sequencing library was produced from this total RNA using the ScriptSeq complete plant leaf kit (Illumina, USA) following the manufacturer’s instructions. The indexed library was sequenced on a MiSeq instrument (Illumina) using a 600-cycle V3 kit. The resulting 707,794 paired reads were 3′ trimmed to a quality score of 20 using Sickle in paired-end mode (8) and assembled using Trinity v2 using 99 Gb of access memory (AM) and 64 central processing units (CPUs) (9), and the resulting contigs were compared to the GenBank nonredundant (nr) and nucleotide databases using BLAST+ (10). Reads of viral origin were extracted using the extract reads function in MEGAN (11). A contig 6,539 nucleotides (nt) long assembled by comparison with other genomes constituted a complete PVT-Bol genome minus its poly(A) tail. PVT-Bol RNA had three open reading frames (ORFs), which encoded a replication-associated protein, a movement protein, and a coat protein. There was an 80 to 81% nt identity between PVT-Bol and the complete genomic sequences of PVT-Type (GenBank accession number NC_011062) and PVT-Jap (GenBank accession number AB697482), isolated in Japan from a Peruvian potato (7, 12). The proposed species demarcation criterion for genus Tepovirus members is <80% coat protein or polymerase amino acid identity (13). The predicted coat protein amino acid sequence identities between PVT-Bol and the 12 other PVT isolates with complete genomic sequences in GenBank (accession numbers JQ316469, EU835973, AB697482, JQ394882, JF297562, JQ394883, JQ394886, JQ407804, JQ394884, JQ394885, JQ407083, and JQ407085), which have 95 to 99% amino acid (aa) identities between themselves, are 93 to 94% identical. Similarly, the predicted polymerase amino acid sequence identities between PVT-Bol and these PVT isolates (which have 97 to 99% aa identities between themselves) are 91 to 92% identical. These sequence differences demonstrate that PVT-Bol is distinct from all PVT isolates sequenced previously but belongs within the same species.

### Data availability.

The PVT-Bol isolate sequence was deposited in GenBank under accession number MH680825. Raw data were deposited in the SRA under accession number SRS3798126, part of BioProject number PRJNA491634.
